# Recognition of terminal buds of densely-planted Chinese fir seedlings using improved YOLOv5 by integrating attention mechanism

**DOI:** 10.3389/fpls.2022.991929

**Published:** 2022-10-10

**Authors:** Zhangxi Ye, Qian Guo, Jiahao Wei, Jian Zhang, Houxi Zhang, Liming Bian, Shijie Guo, Xueyan Zheng, Shijiang Cao

**Affiliations:** ^1^College of Forestry, Fujian Agriculture and Forestry University, Fuzhou, China; ^2^College of Forestry, Nanjing Forestry University, Nanjing, China; ^3^Co-Innovation Center for Sustainable Forestry in Southern China, Nanjing Forestry University, Nanjing, China; ^4^Key Laboratory of Forest Genetics & Biotechnology of the Ministry of Education, Nanjing Forestry University, Nanjing, China; ^5^Key Laboratory of State Forestry and Grassland Administration for Soil and Water Conservation in Red Soil Region of South China, Fuzhou, China; ^6^Cross-Strait Collaborative Innovation Center of Soil and Water Conservation, Fuzhou, China; ^7^Seed and seedling department, Yangkou State-owned Forest Farm, Nanping, China

**Keywords:** UAV-based remote sensing, Chinese fir seedling, YOLOv5 algorithm, deep learning, attention machanism

## Abstract

Accurate and timely information on the number of densely-planted Chinese fir seedlings is essential for their scientific cultivation and intelligent management. However, in the later stage of cultivation, the overlapping of lateral branches among individuals is too severe to identify the entire individual in the UAV image. At the same time, in the high-density planting nursery, the terminal bud of each seedling has a distinctive characteristic of growing upward, which can be used as an identification feature. Still, due to the small size and dense distribution of the terminal buds, the existing recognition algorithm will have a significant error. Therefore, in this study, we proposed a model based on the improved network structure of the latest YOLOv5 algorithm for identifying the terminal bud of Chinese fir seedlings. Firstly, the micro-scale prediction head was added to the original prediction head to enhance the model’s ability to perceive small-sized terminal buds. Secondly, a multi-attention mechanism module composed of Convolutional Block Attention Module (CBAM) and Efficient Channel Attention (ECA) was integrated into the neck of the network to enhance further the model’s ability to focus on key target objects in complex backgrounds. Finally, the methods including data augmentation, Test Time Augmentation (TTA) and Weighted Boxes Fusion (WBF) were used to improve the robustness and generalization of the model for the identification of terminal buds in different growth states. The results showed that, compared with the standard version of YOLOv5, the recognition accuracy of the improved YOLOv5 was significantly increased, with a precision of 95.55%, a recall of 95.84%, an F1-Score of 96.54%, and an mAP of 94.63%. Under the same experimental conditions, compared with other current mainstream algorithms (YOLOv3, Faster R-CNN, and PP-YOLO), the average precision and F1-Score of the improved YOLOv5 also increased by 9.51-28.19 percentage points and 15.92-32.94 percentage points, respectively. Overall, The improved YOLOv5 algorithm integrated with the attention network can accurately identify the terminal buds of densely-planted Chinese fir seedlings in UAV images and provide technical support for large-scale and automated counting and precision cultivation of Chinese fir seedlings.

## 1 Introduction

*Cunninghamia lanceolata* (Lamb.) Hook is one of southern China’s most critical timber species for afforestation. It plays an essential role in forest carbon sink in China, for its cultivation area accounts for 20% of the national plantation forest area, and its stock volume accounts for 25% of the national plantation volume (Liu et al., 2022). Due to the increasing demand for improved seedlings for reforestation in harvested sites, the scale of Chinese fir seedling cultivation has been expanding recently. In this context, it is critical to attaining the accurate number and cultivation density of Chinese fir seedlings, which can provide essential support for scientific cultivation and intelligent management, such as thinning time, precise seedling determination, and water, fertilizer, and light management (Mateen and Zhu, 2019; Dorbu et al., 2021). Moreover, it is vital in disease prevention and control, seedling emergence rate estimation, and nursery asset valuation (Chen et al., 2017; Shen et al., 2020).

Traditionally, the method of determining the number of Chinese fir seedlings is based on statistics by manually calculating the number of sampled plots. This method is inefficient and may also lead to significant errors, which is limited in its large-scale application (Marques et al., 2019; Mohan et al., 2021). With the rapid development of spatial information technology, the means of monitoring by remote sensing has gradually attracted widespread attention. However, due to the low spatial resolution, the traditional satellite-based remote sensing cannot observe small targets such as Chinese fir seedlings (Zhu et al., 2021b), so it is challenging to accurately identify the seedlings with it. In recent years, the emergence of the UAV remote sensing platform has brought an opportunity to solve this problem. This platform, flying at a height much lower than the satellites, can acquire images with super-spatial resolution (pixel size<10cm) (Colpaert, 2022). Therefore, the UAV-based images can clearly display the structural features (shape, size, and texture) of ground objects (Bhandari et al., 2018; Osco et al., 2021), which to a large extent overcomes the limitations of traditional remote sensing, making the extraction of tiny targets possible (Yin et al., 2021). The terminal bud is an integral part of the morphological structure of Chinese fir seedlings. Because of its apparent characteristics of growing upward, the terminal bud can be a symbolic feature for detecting a single Chinese fir seedling. The UAV-based remote sensing can be a new potential means for acquiring accurate numbers and cultivation density of Chinese fir seedlings by detecting their terminal buds.

Deep learning algorithm based on artificial intelligence is a new field of machine learning. Thanks to its robust feature extraction capability, this algorithm has more tremendous advantages than traditional machine learning algorithms in processing massive high-dimensional data (Zhu et al., 2017; Ball et al., 2018; Cheng et al., 2020; Changhui et al., 2021). The successful application of deep learning technology in computer vision provides essential technical support for the intelligent extraction of plant information in agriculture and forestry (Haq et al., 2021; Bian et al., 2022; Ye et al., 2022). Among them, the YOLO series algorithms are the most widely used target detection algorithms (Tong et al., 2020; Wu et al., 2020) Many scholars have improved them for different application scenarios to improve detection accuracy and efficiency further. Lv et al. (2022) proposed an improved YOLOv3 model and used it to detect crop pests in natural agricultural environments by combining it with image enhancement; Wang et al. (2022) proposed an improved lightweight YOLOv4-based model to detect dense plums in orchards; Zhang et al. (2022) proposed an improved YOLOv5-CA model for real-time control of disease transmission on grapevines in precision viticulture. However, the current standard YOLO networks are designed for objects with large size and low density (Zhu et al., 2021b), while the terminal buds of Chinese fir seedlings in UAV images are small target objects. Their area ratio in the image is minimal and has characteristics of high density, sever overlapping, and occlusion, so they are prone to false detection and missed detection. Meanwhile, the morphology of lateral branches of Chinese fir seedlings is similar in shape to the terminal buds of Chinese fir seedlings The number and density of lateral branches are both much larger than that of terminal buds, which makes their background in UAV images very complicated. Moreover, terminal buds of densely-planted seedlings vary in size, including micro, small, medium and large ones, due to the different growth states of individuals, which further increases the difficulty of detection. Therefore, the direct application of the existing target detection network of the YOLO algorithm in detecting of the terminal buds of Chinese fir seedlings from UAV images will have a significant error.

In this study, we propose a method for recognizing the terminal bud of densely-planted Chinese fir seedling based on an improved YOLOv5 algorithm using a UAV RGB image. The method is based on the standard YOLOv5 algorithm which is improved by adding micro-scale prediction heads, new connections between backbone and neck networks, and introducing an attention mechanism module consisting of a Convolutional Block Attention Module (CBAM) and an Efficient Channel Attention (ECA). The specific objectives of this study are: 1) to evaluate the accuracy, stability, and efficiency of the improved YOLOv5 algorithm that integrates attention mechanism in detecting terminal bud of Chinese fir seedlings; 2) to compare the performance of the improved YOLOv5 algorithm with current mainstream target detection algorithms (YOLOv3, Faster R-CNN, and PP-YOLO).

## 2 Materials and methods

### 2.1 The study area

**Figure 1:**
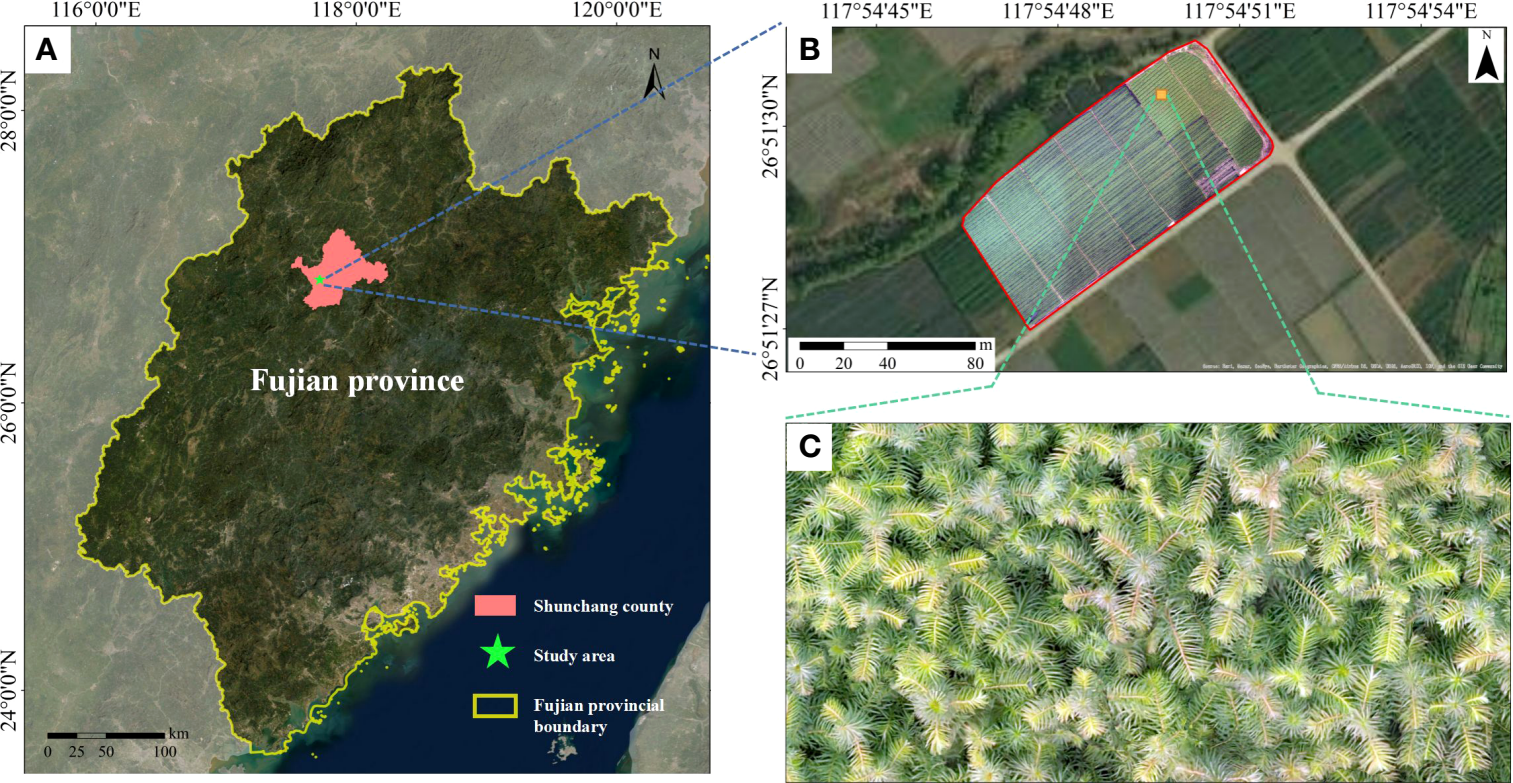
Study area. **(A)** Geographical location of the study area, **(B)** Overview of study Area, **(C)** Local details of the study area.

The study area (117°40′E, 26°50′N) is located at the breeding base of Chinese fir seedlings in Yangkou state-owned forestry farm in Shunchang County, Fujian Province, China (**Figure 1**). This area is characteristic of the mid-subtropical maritime monsoon climate, with an annual average temperature of 18.5°C, an annual average rainfall of 1756 mm, an annual sunshine duration of about 1740 hours, and a frost-free period of about 305 days. The Chinese fir seedlings cultivated include 14 excellent asexual lines and three generations of live seedlings, such as “Yang 020”, “Yang 062” and “Yang 003”, of which the seedling density is 50~55 thousand per mu and the qualified seedlings are 40-45 thousand plants. The county’s total nursery area reaches 11.13 ha, and the seedlings are usually planted in mid-December every year.

### 2.2 Data collection and dataset construction

**Figure 2:**
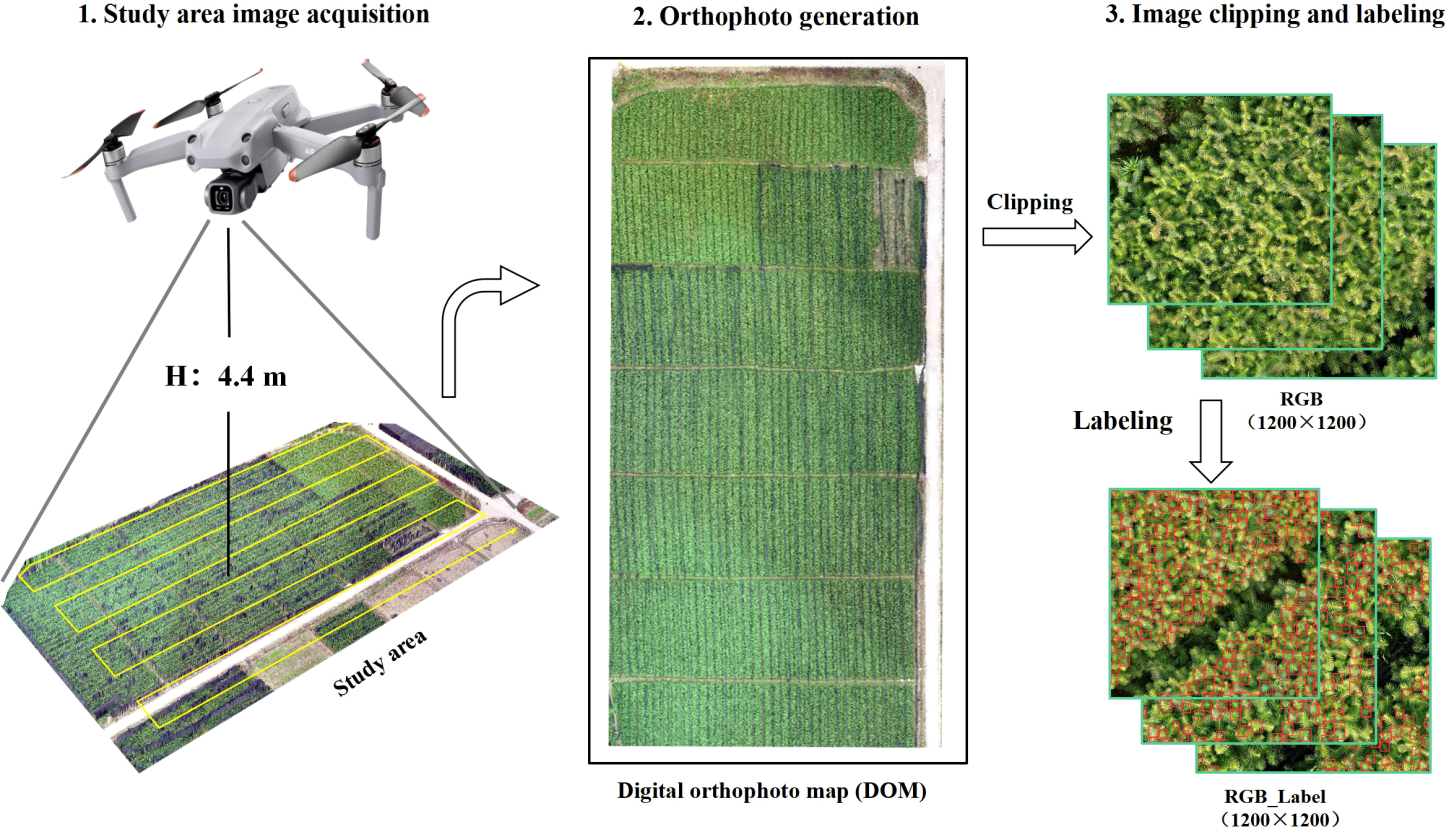
Flow of image data acquisition.

On November 28th, 2021, a DJI Air2S drone (DJI Technology Co., Ltd., Shenzhen, China) collected visible light images of Chinese fir seedlings in the research area (**Figure 2**). The drone has a visible-light sensor (1 inch) with 20 million pixels (pixel size of 2.4 μm) and a camera equivalent focal length of 22 mm. Rainbow software was used for route planning. The flight altitude was set to 4.4 m, and the overlap rate in the side direction and the heading were set to 80%. Meanwhile, the shutter speed was set to 1/320s to avoid blurred images caused by the slow shutter speed during the drone’s movement. A total of 1935 photos were acquired, and orthophotos were generated by stitching with Pix4d software (Pix4D China Technology Co., Ltd, Shanghai, China).

Due to the vast data of UAV images and the limited performance of computer hardware, it is hard to input the images into the YOLO network framework for processing at one time. Therefore, it is necessary to first crop the orthophoto into sub-blocks with a size of 1200×1200 pixels. Then, a rectangular box outside the target terminal bud was drawn using the image data labeling software (LabelImg) to label the terminal bud manually. To ensure that the rectangular box contains as little background as possible, it is drawn according to the minimum rectangular box principle. Finally, the labeled sample data were divided into the training set, validation set, and test set according to the ratio of 7:2:1 to construct the terminal bud dataset of the Chinese fir seedling by the VOC format. The dataset of the Chinese fir seedling terminal bud consists of 173 images with a size of 1200*1200, and contains a total of 25,938 terminal bud annotation boxes with different sizes, occlusions, defects, angles, and illumination; the aspect ratio of the annotation box is between 0.8 and 1.6, the overlap is between 0 and 0.25, and the number of pixels for length and width is between 12 and 48.

### 2.3 Data augmentation

#### 2.3.1 Data augmentation of training set

**Figure 3:**
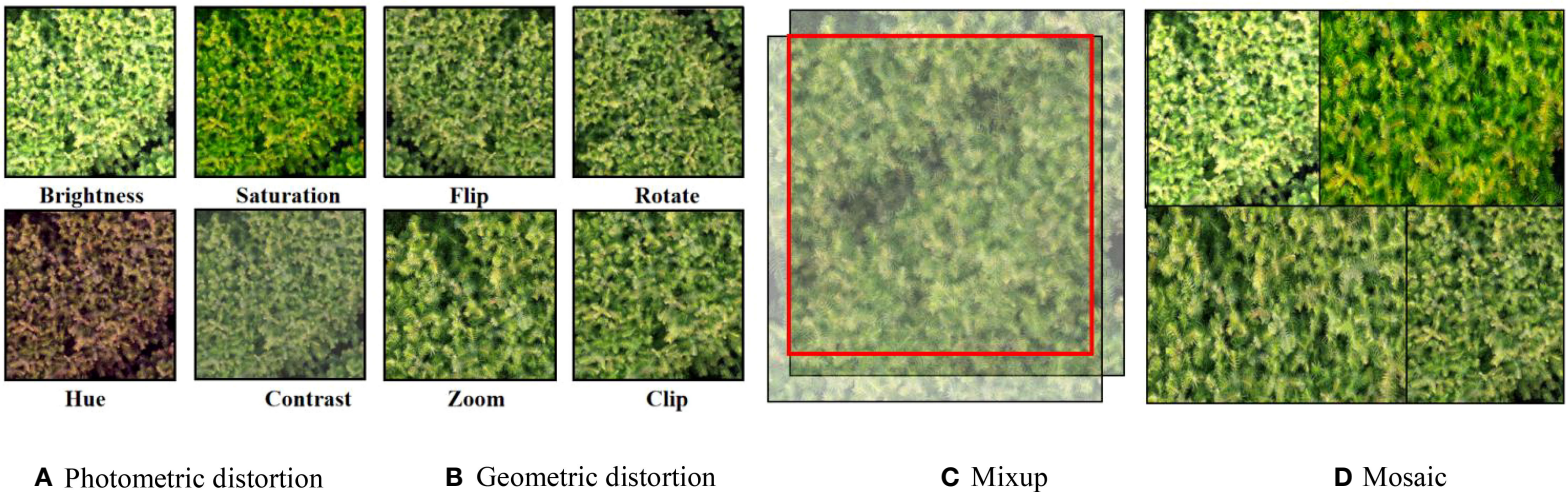
Data augmentation. **(A)** Photometric distortion, **(B)** Geometric distortion, **(C)** Mixup, **(D)** Mosaic.


Data augmentation is an effective means to expand the training dataset, which can enhance the robustness and generalization of the model under uncertain factors such as different illumination angles, growth states, and fuzzy occlusions (Shorten and Khoshgoftaar, 2019). Traditional data augmentation methods include two main types: global photometric distortion and geometric distortion. Global photometric distortion mainly performs random image adjustment of hue, saturation, brightness, and contrast, while global geometric distortion performs random scaling, cropping, flipping, and rotating operations on standard images. This study introduces novel augmentation methods based on the two traditional augmentation methods mentioned above (**Figure 3**). In recent years, research in computer vision has shown that data augmentation by fusing multiple images can improve the target detection performance of models in complex scenes. Mixup (Zhang et al., 2017), Cutmix (Yun et al., 2019), and Mosaic (Bochkovskiy et al., 2020) are the three most commonly used data augmentation methods for multi-image fusion. The Mixup fusion method is a computer vision algorithm for image mixing enhancement. It expands the training data set by fusing different images. Its principle is to randomly select two samples from the images contained in training set for random weighted summation, and its sample labels are also weighted and summed accordingly. The Mixup principle is shown in equations 1~3.

(1) λ=Beta(α,β)

(2) $$mixed\_batch_{x}=\lambda \times batch_{x1}+(1-\lambda )\times batch_{x2}$$

(3) $$mixed\_batch_{\text{y}}=\lambda \times batch_{\text{y}1}+(1-\lambda )\times batch_{y2}$$

Note: β refers to the beta distribution, *α, λ*, *mixed_batch_x_* is the mixed sample, and *mixed_batch_y_* is the label corresponding to the mixed sample.

Unlike the traditional method of using 0-value pixels to occlude the image randomly, the Cutmix method randomly uses a particular area of an image to complete the occlusion of the image. As an improved version of Cutmix, Mosaic randomly selects four images for cropping and mosaicking, which significantly enriches the background features of the training objects and enables the model to perform well in complex scenes. Therefore, this study introduces two new fusion methods, Mixup, and Mosaic, into the data augmentation processing.

#### 2.3.2 TTA and WBF

Test Time Augmentation (TTA) is a method for extending test datasets, which can effectively improve the performance of deep learning models (Moshkov et al., 2021). Its working principle is that in the inference (prediction) stage, the standard image is first scaled, flipped, and rotated. Then the trained model is used to predict the different versions of each image in the test data set. Finally, the different augmentation results of the same image are analyzed together to obtain the result with the slightest error.

**Figure 4:**
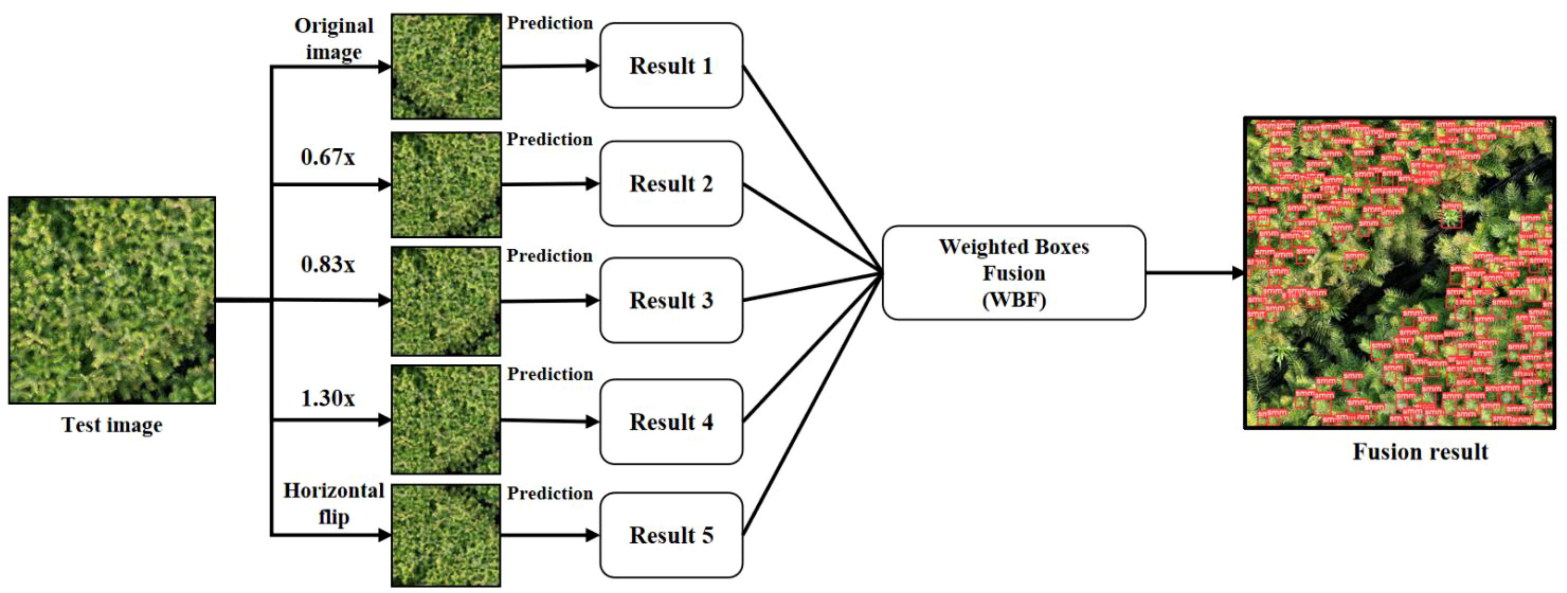
Schematic diagram of test time augmentation (TTA).

**Figure 5:**
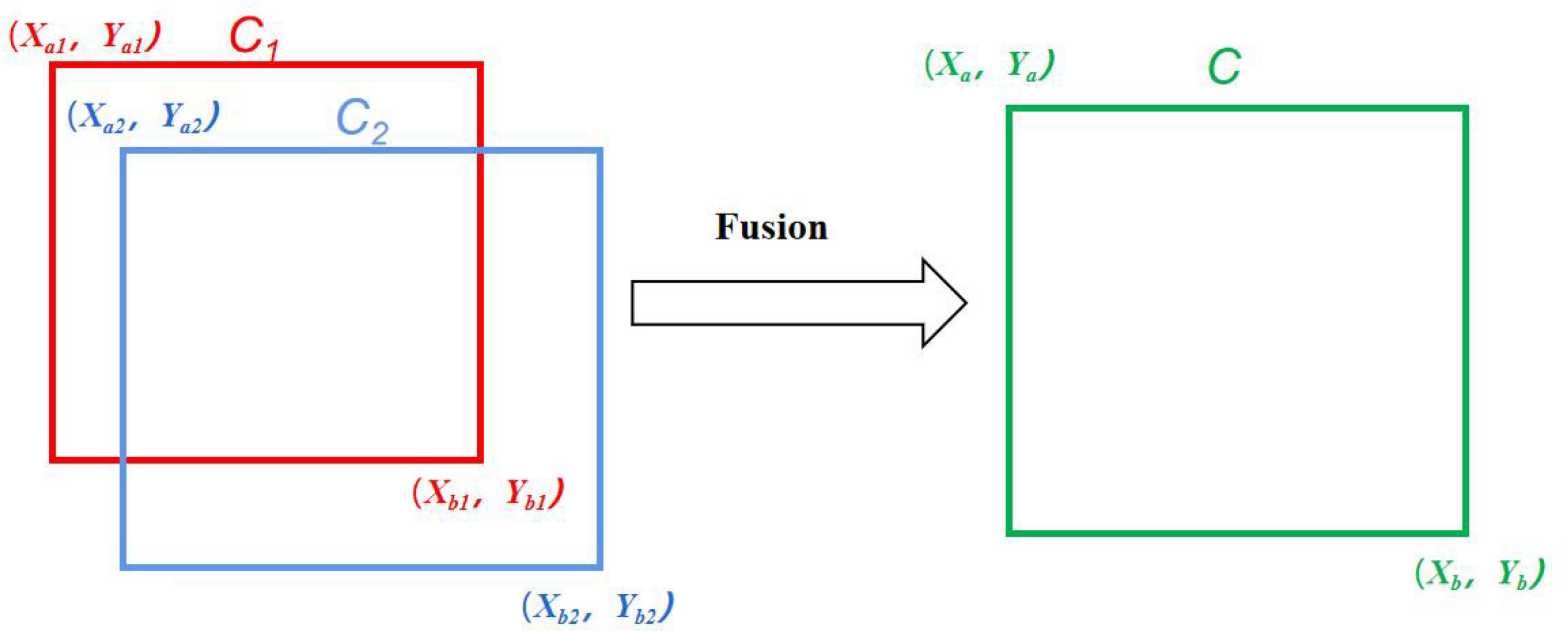
Principle of the weighted box fusion algorithm.

In the target detection task, better results can be obtained by fusing multiple predictions. There are three main algorithms commonly used at present (**Figure 4**): Non-Maximum Suppression (NMS) (Neubeck and Van Gool, 2006), Soft-NMS (Bodla et al., 2017), and Weighted Boxes Fusion (WBF) (Solovyev et al., 2021). The NMS method is based on the principle that when there are multiple overlapping boxes in the prediction result and the Intersection over Union (IoU) ratio is greater than a certain threshold, they are considered to belong to the same object. This method only retains the highest confidence box and deletes the others. Soft-NMS is an improved algorithm based on NMS, which sets a decay function for the confidence of adjacent prediction boxes based on the IoU value instead of setting their confidence to 0 and deleting them. The weighted box fusion algorithm used in this paper (**Figure 5**) works differently from the previous two. WBF calculates fusion weights according to the confidence levels of different terminal bud prediction boxes generated after TTA augmentation. The coordinates of multiple prediction boxes are fused to serve as the final prediction boundaries of the terminal buds of Chinese fir seedlings.

Among them, in the process of generating a new box by fusion, the shape and position of the new boxes are more inclined towards the box with a larger weight, as shown in **Equation 4**.


(4) $$X_{\text{a}}=\frac{\sum_{i=1}^{\text{Z}}{\text{ C}}_{i}\times X_{ai}}{\sum_{i=1}^{\text{Z}}{\text{ C}}_{i}},Y_{\text{a}}=\frac{\sum_{i=1}^{\text{Z}}{\text{ C}}_{i}\times {\text{Y}}_{ai}}{\sum_{i=1}^{\text{Z}}{\text{ C}}_{i}}=\frac{\sum_{i=1}^{\text{Z}}{\text{ C}}_{i}\times {\text{X}}_{bi}}{\sum_{i=1}^{\text{Z}}{\text{ C}}_{i}},Y_{b}=\frac{\sum_{i=1}^{\text{Z}}{\text{ C}}_{i}\times Y_{bi}}{\sum_{i=1}^{\text{Z}}{\text{ C}}_{i}},C=\frac{\sum_{i=1}^{\text{Z}}\;C_{i}}{\text{Z}}$$

**Figure 6:**
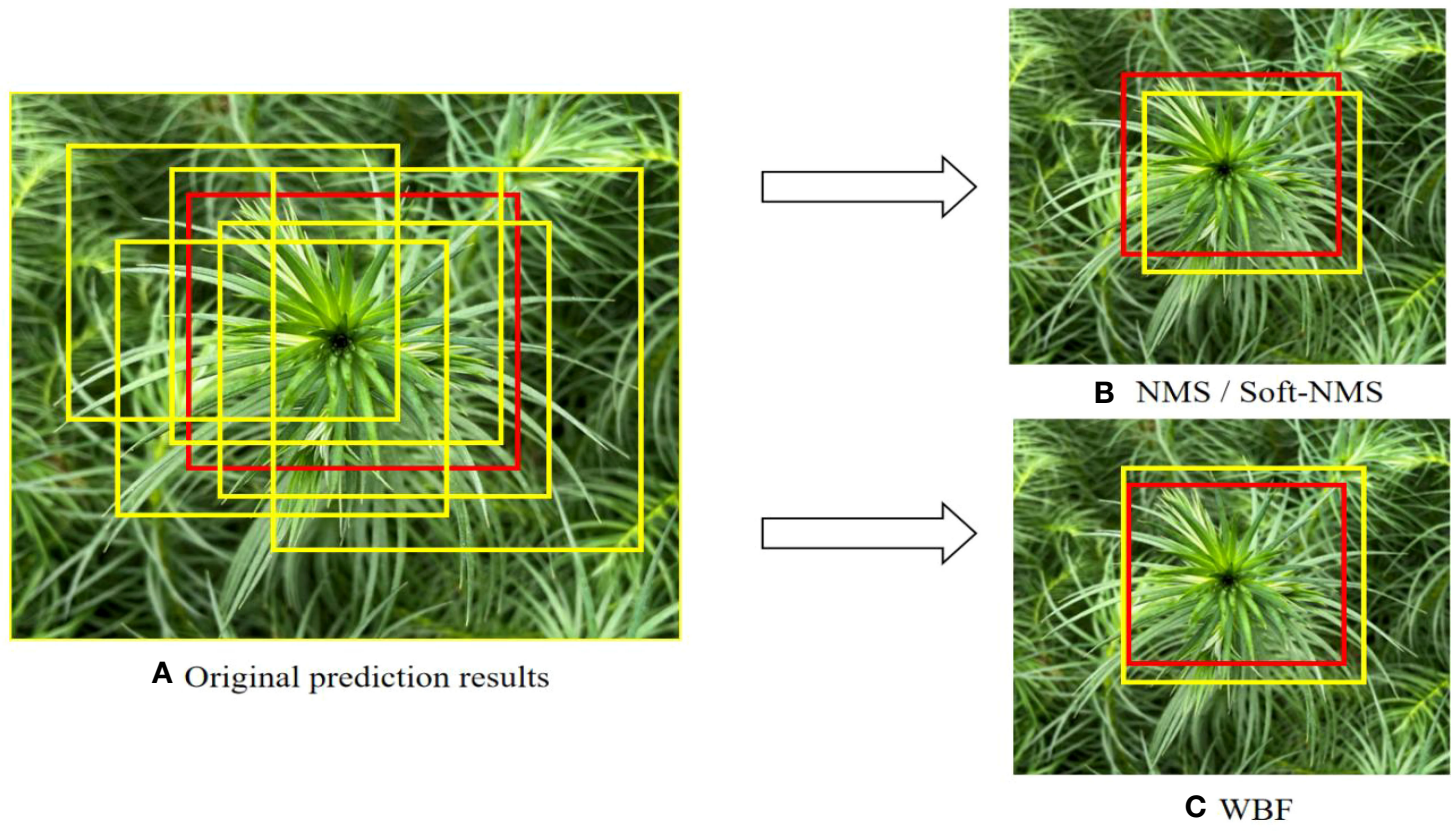
Schematic diagram of Non-maximum inhibition (NMS), Soft-NMS, and Weighted Box Fusion (WBF). The red box is the real boundary box of the terminal bud of Chinese fir seedlings, and the yellow box is the model prediction box. **(A)** Original prediction results, **(B)** NMS / Soft-NMS, **(C)** WBF.

Where *X_a_, Y_a_, X_b_, Y_b_* are the coordinates of the top-left and bottom-right vertices of the fused predictors, respectively; C is the confidence level of the fused predictors; *X_ai_, Y_ai_, X_bi_, Y_bi_* are the coordinates of the top-left and bottom-right vertices of the participating predictors; *C_i_* is the confidence level corresponding to each predictor, and *Z* is the number of participating predictors. Both NMS and Soft-NMS will eliminate some of the prediction boxes, while WBF fuses all the prediction boxes to form the final result, reducing the model’s prediction error to some extent. Therefore, this paper uses the WBF algorithm. It can be seen from **Figure 6** that the actual application effect and performance of the WBF algorithm are significantly improved compared with both NMS and Soft-NMS.

### 2.4 Attentional mechanisms

#### 2.4.1 CBAM attention module

Since the importance of the features of the target object in each channel is different, and the importance of pixels at various locations in each channel also varies, only by considering these two different levels of importance simultaneously can the model recognize the target object more accurately. Therefore, we insert the Convolutional Block Attention Module (CBAM) into the neck of the modified YOLOv5 (Woo et al., 2018), which is a simple and effective lightweight attention module with a dual attention mechanism, i.e., a Channel Attention Module (CAM) and a Spatial Attention Module (SAM).

**Figure 7:**
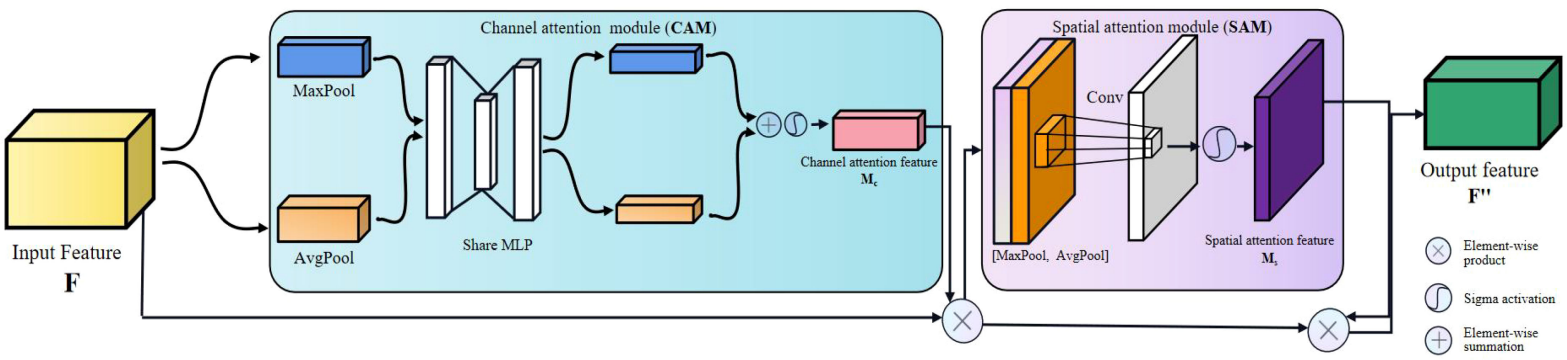
Structure diagram of the convolutional block attention module (CBAM).

CBAM generating attention can be divided into two parts (**Figure 7**). First, a network intermediate feature map F ∈ R (C × H × W) is given as input, where C represents the number of channels, H and W denote the length and width of the feature map in pixels. Different channels perform global maximum pooling and mean pooling on the input feature map **F**. The two one-dimensional vectors after pooling are sent to a multi-layer perceptron (MLP) composed of a hidden layer for combining operations. Second, the *Sigma* function adds and activates the corresponding elements to generate one-dimensional channel attention. Finally, the channel attention feature map **Mc** is multiplied one by one with the elements of the input feature map **F** to obtain the feature map **F’** weighted in the channel dimension; the generated feature map **F’** is input to the spatial attention module, and the global maximum pooling and mean pooling are performed by spatial dimension. The two feature maps generated by pooling (the number of channels is 1) are concatenated and activated by the sigmoid function to generate the spatial attention feature map **Ms**, which is then multiplied by **F’** by element, and finally an attention-weighted feature map with two-dimension (channel and spatial) is obtained.

#### 2.4.2 ECA attention module

**Figure 8:**
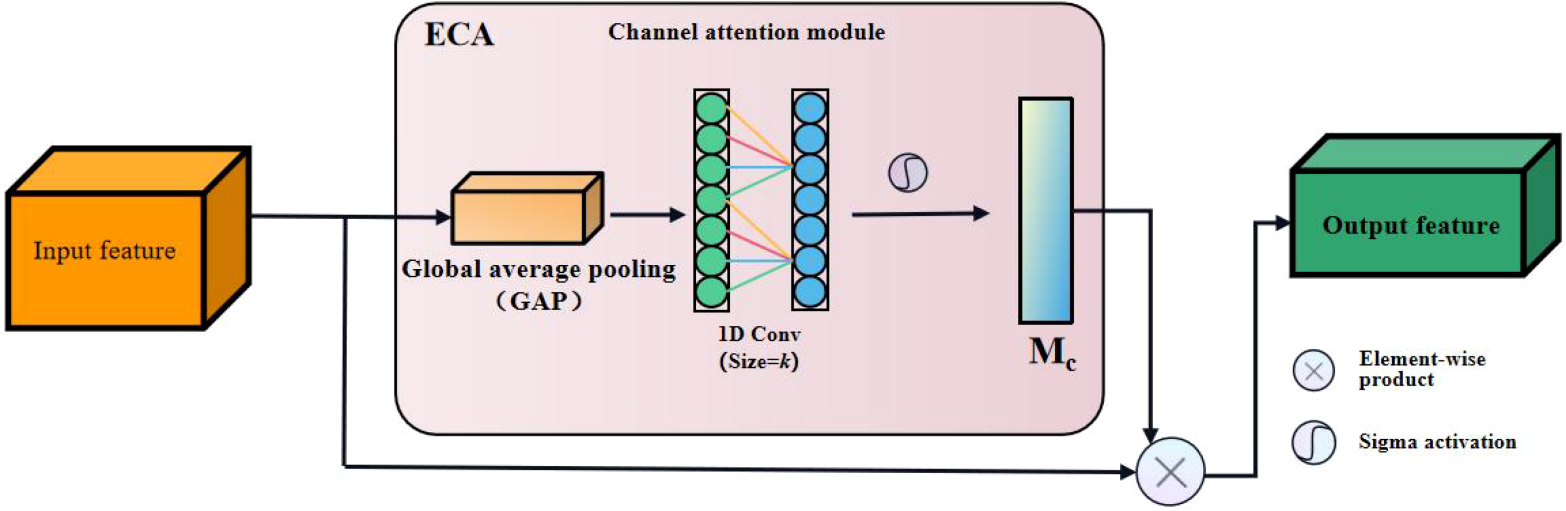
Structure diagram of the Efficient Channel Attention (ECA) module.

The ECA (Efficient Channel Attention) attention mechanism is a channel attention mechanism based on the SE (Squeeze and Excitation) attention module with lightweight improvements(Wang et al., 2020). Its structure is shown in **Figure 8**.

Since the channel attention module (CAM) in CBAM reduces the spatial dimension of feature maps through global average pooling and max pooling operations to obtain nonlinear correlation information among different channels, the process of CBAM to control the complexity of the model through dimensionality reduction will have a side effect on the interaction information among the channels, resulting in a decrease in the prediction accuracy. Yet the ECA model can solve this problem. The ECA module captures nonlinear information across channels by fast one-dimensional convolution instead of fully connected layers, allowing the network to learn channel attention more efficiently while reducing the amount of computation. The size of the convolution kernel of the one-dimensional convolution in **Figure 8** is k, which represents the coverage of cross-channel information, i.e., the current channel and the adjacent k channels are jointly involved in predicting channel attention. The ECA module adopts an adaptive approach to determine k, as shown in **Equation 5**.


(5) $$k=f(c)=\left|\frac{\log_{2}C+b}{a}\right|odd$$

Note: Where, *c* is the channel dimension, | *x* | represents the nearest odd number to *x*.

### 2.5 Target detection network structure of improved YOLOv5

YOLOv5 is currently recognized as one of the most effective target detection models, which is not only highly accurate and fast but also highly flexible (Luo et al., 2022). The network structure of YOLOv5 is mainly divided into the backbone part for feature extraction, the neck part for feature fusion, and the head part for target detection (Zhang et al., 2021; Xue et al., 2022). The backbone module adopts Cross Stage Partial Network (CSPNet) and Spatial Pyramid Pooling-Fast (SPPF) to extract input image feature and transmit them to the neck module. The neck module uses the Path Aggregation Network (PANet) to generate a feature pyramid and bi-directionally fuse low-level spatial features with high-level semantic features to enhance the detection ability of objects at different scales. The head module is responsible for generating target prediction boxes to determine the category, coordinates, and confidence level of the detected object (Wen et al., 2021). Its network contains four network structures of different sizes (YOLOv5s, YOLOv5m, YOLOv5l, and YOLOv5x), thus allowing the user to choose the appropriate model according to their actual needs (Song et al., 2021). Since this research mainly considers the accuracy problem when selecting the recognition algorithm, and does not require high real-time requirement of the algorithm, the YOLOv5x network with the deepest network depth and the widest feature map width is selected.

**Figure 9:**
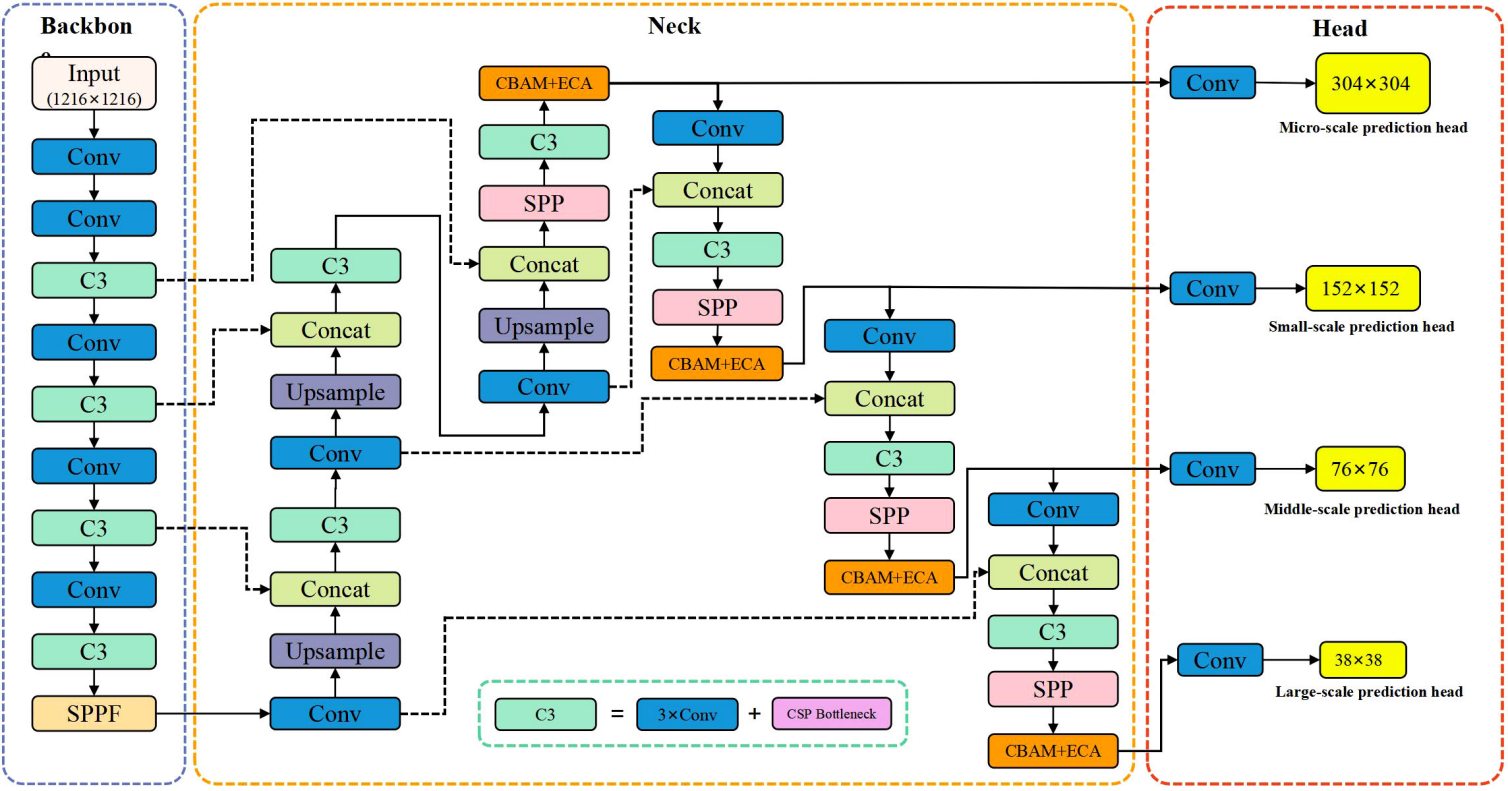
Improved YOLOv5 network structure diagram.

Although YOLOv5 has good detection and inference performance, it still has certain limitations if it is directly applied to detect of dense small targets such as terminal buds of Chinese fir seedlings. To more accurately detect the terminal buds of Chinese fir seedlings from UAV images, this study optimized and improved the YOLOv5 network and proposed an improved YOLOv5 that integrates the attention mechanism. The specific network structure is shown in **Figure 9**.

**Figure 10:**
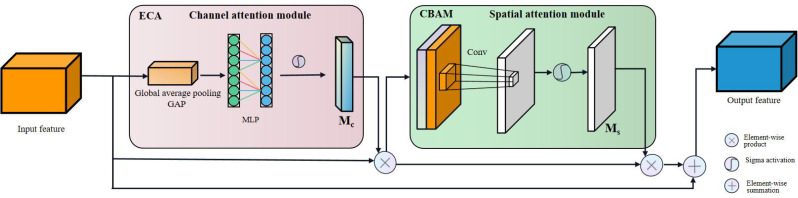
The structure of the attention modules.

This paper has the following four improvements to the standard YOLOv5 architecture:

(1) To improve the detection performance of YOLOv5 for tiny terminal buds of Chinese fir seedlings, a new Micro-scale Predictive Head (MSPH) is added to the head section of YOLOv5, which is derived from 4-fold downsampling to generate a larger feature map (size 304 × 304). Compared with 8, 16, and 32 times downsampling detection heads of the standard YOLOv5 model, this micro-scale head can utilize higher-resolution feature maps in the shallow layers to capture more delicate feature information of the tiny terminal buds. The improved head section has four prediction heads of different scales, which can be used to detect tiny, small, medium, and large targets, respectively, which can effectively improve the model’s ability to detect terminal buds of different sizes.

(2) Based on the idea of a residual network, a new network connection is added (as shown by the dashed arrow in **Figure 9**). By introducing the feature information of the backbone network into the feature fusion layer of the neck network, the back propagation of the gradient can be strengthened, the phenomenon of gradient decay can be avoided, and the loss of feature information of small objects can be reduced.

(3) The attention module was added to the neck feature fusion layer to highlight the critical information of the terminal buds of Chinese fir seedlings (**Figure 10**). The new attention module is implemented by a combination of CBAM and ECA-Net, where the ECA-Net module implements channel attention and spatial attention is derived from the original CBAM module. The ECA-Net module first learns the features processed by global average pooling through one-dimensional convolution. It multiplies the updated channel weights with the input feature map to generate a new feature map. Secondly, the spatial attention module (SAM) in the CBAM module takes the feature map generated by the ECA-Net module as input, generates a spatial attention feature map, and adds it to the original feature map to simulate the residual block structure. Finally, a Relu activation function is applied to the summed feature maps to generate feature maps with a dual attention mechanism. By integrating the CBAM-ECA attention module in the network structure of the neck, the model makes it less susceptible to the complex background and can obtain more important feature information of the terminal buds from the complex background. This can effectively increase the model’s robustness and improve its recognition ability.

(4) The TTA multi-scale test method is introduced in the image inference prediction stage, i.e., data augmentation is performed on the images of the test dataset. The test images are scaled (3 different multiples) and horizontally flipped to obtain six images of different scales. The model can achieve better prediction performance and reduce generalization errors by testing these six images and fusing the prediction results using the WBF algorithm.

**Figure 11:**
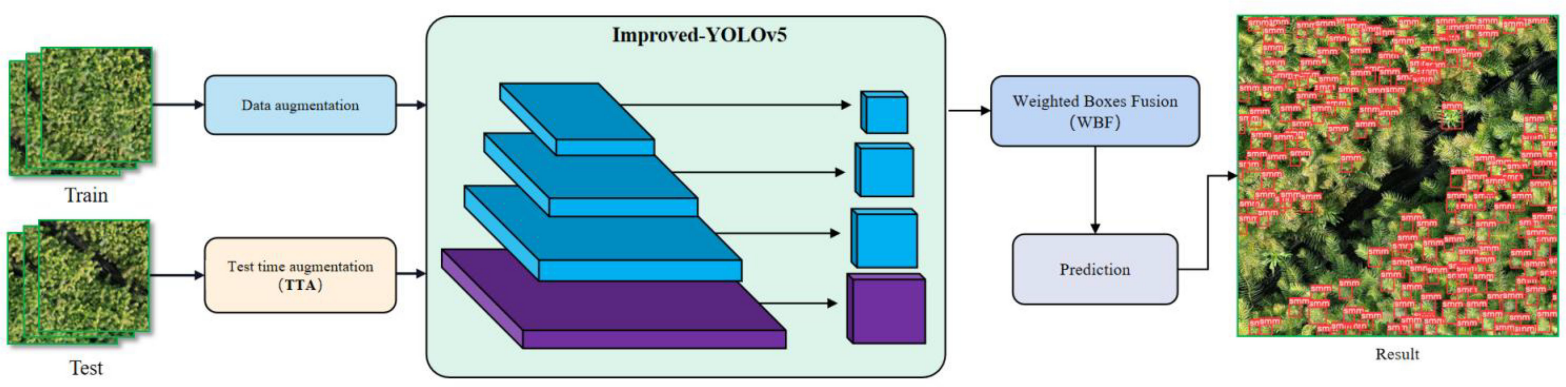
Improved YOLOv5 workflow chart.

The workflow of the improved YOLOv5 is shown in **Figure 11**. In the data preprocessing stage, the data augmentation process is mainly performed for MixUp, Mosaic, photometric distortion and geometric distortion, and the enhanced image dataset will be input into the improved YOLOv5 network for training. In the prediction stage, the model is firstly tested with TTA multiscale, and the test images are scaled by 1.30 times, 0.83 times and 0.67 times. Then the images are flipped horizontally, and finally, the enhanced test images are input into the improved YOLOv5 network and the TTA prediction results are fused using the WBF algorithm to obtain the final results.

### 2.6 Accuracy evaluation

Five metrics are used to evaluate the model accuracy: precision, recall, F1-Score, Mean Average Precision (mAP), and Frames Per Second (FPS). If the intersection over union (IoU) ratio between the prediction box and the manually labeled bounding box of the terminal buds of Chinese fir seedlings is greater than 0.5, the prediction box is marked as correctly identifying the sample TP; otherwise, it is marked as FP. If the manually labeled bounding box of the terminal buds of Chinese fir seedlings has no matching prediction box, it is marked as FN. Precision evaluates the number of true positive cases in the predicted positive case results from the perspective of model prediction results, i.e., the accurate number of terminal buds predicted by the model. Recall is from the perspective of true data set samples, describing the number of true positive cases in the test set identified by the model, i.e., the number of true terminal buds correctly determined by the model. F1-Score is the weighted summed average of the precision and recall; the higher the value, the higher the robustness of the model. The mAP is often used as an indicator to measure detection accuracy in target detection. Compared with precision and recall, it can better reflect the model’s global performance. The equation for calculating the above indicators is as follows:


(8) $$\text{Precision}=\frac{\text{TP}}{\text{TP}+\text{FP}}\times 100\%$$

(9) $$\text{Recall}=\frac{\text{TP}}{\text{TP}+\text{FN}}\times 100\%$$

(10) $$F1-\text{Score}=2\times \frac{\text{Precision}\times \text{Recall}}{\text{Precision}+\text{Recall}}\times 100\%$$

(11) $$\text{mAP}=\sum_{K=i}^{N}P(k)\Delta \text{R}(k)$$

Note: Where, *N* represents the number of IoU threshold, *k* is the IoU threshold, *P (k)* is the precision, and *R (k)* is the recall.

## 3 Result

### 3.1 Model training and validation

The hardware and software environment of this experiment is shown in **Table 1**. Since the VisDrone2021 UAV image dataset contains many targets of different sizes, the improved YOLOv5 model was first pre-trained using the VisDrone2021 large dataset. Then the dataset of the terminal buds of Chinese fir seedlings was trained through transfer learning. The Adam optimization algorithm was used in the training process, and the initial learning rate was set to 0.0025. The learning rate was reduced by the Cosine annealing method so that the last iteration’s learning rate decayed to 0.12 of the initial learning rate. Due to the high resolution of the dataset (1200×1200 pixels), only the batch size is set to 16 to prevent GPU memory overflow.

**Table 1:** Experimental software and hardware environment.

Name	Parameters and versions
Central Processing Unit (CPU)	AMD EPYC 7543 32-Core Processor @ 2.8 GHz
Memory (RAM)	64GB
Hard Disk Drive (SSD)	INTEL S3710 (1.2 TB)
Graphics Card (GPU)	NVIDIA A40 (48 GB)
Operating System (OS)	Ubuntu 18.04
Programming Environment (ENVS)	PyTorch 1.11.0+Python 3.9

**Figure 12:**
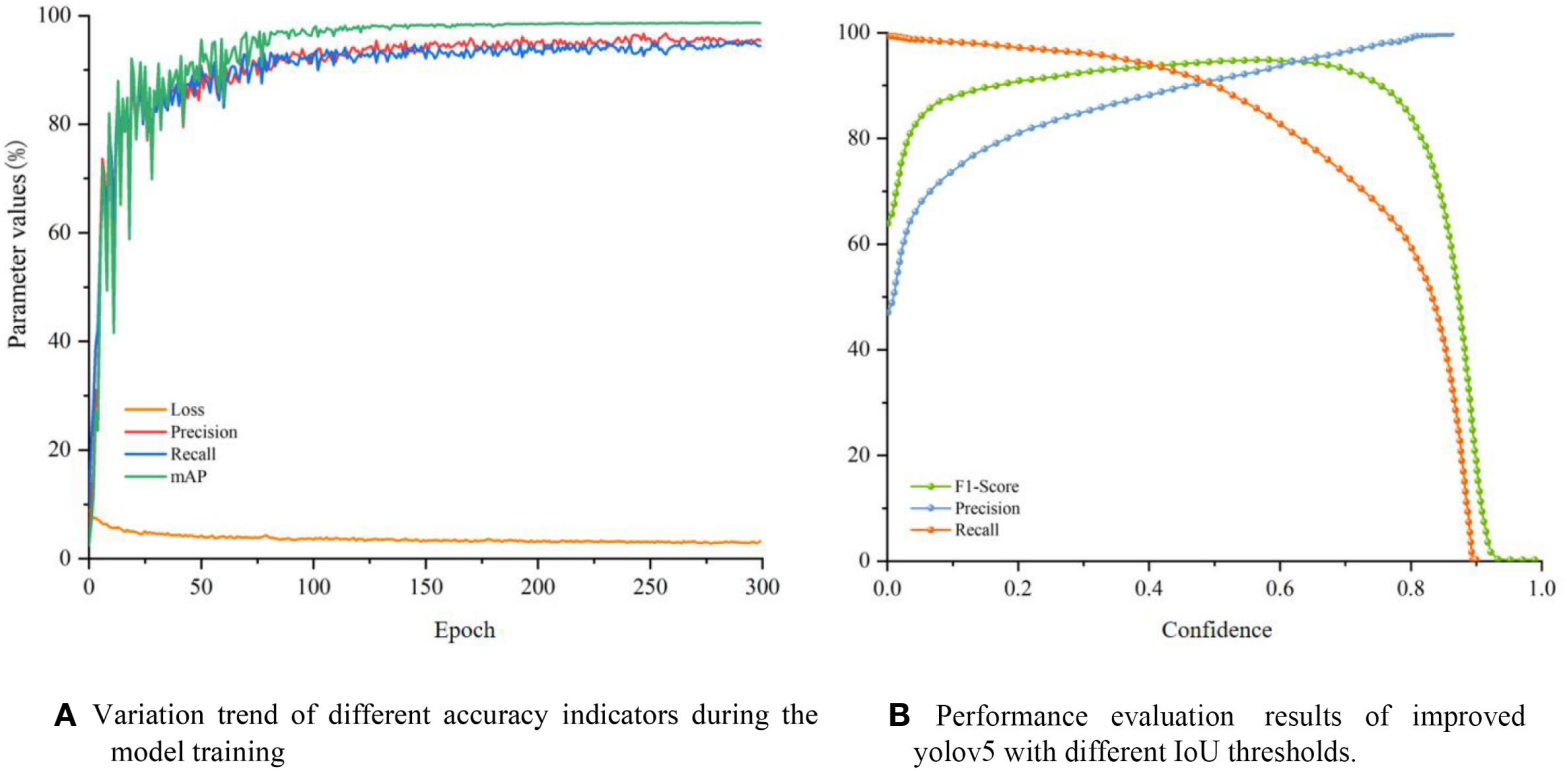
**(A)** Variation trend of different accuracy indicators during the model training, **(B)** Performance evaluation results of improved yolov5 with different IoU thresholds.

The trends of different accuracy indicators in the training process are shown in **Figure 12A**. It can be seen that, in the first 100 iterations of training, the accuracy rate and recall rate of the model increased rapidly, while the loss value decreased rapidly. All the indicators leveled off after 100 iterations, indicating that the model was close to convergence. After 300 iterations of training, the slope of each accuracy indicator curve of the improved model converged to 0, and the loss rate was close to the minimum, indicating that the model had converged. The loss rate is close to the minimum value, indicating that the model has converged, and the training is terminated at this time to prevent overfitting.

It can be seen from the confidence versus accuracy curves (**Figure 12B**) that when the confidence level is greater than 0.15, the accuracy rate of the model is greater than 80%. When the confidence level is less than 0.8, the recall rate is maintained at a fairly high level, and when the confidence is greater than 0.8, the recall rate sharply drops until it approaches 0. When the confidence level is between 0.05 and 0.8, the F1-Score is all greater than 80%, indicating that the improved YOLOv5 has higher accuracy and stability within the large-span confidence interval. The above three indicators show that the improved YOLOv5 model has good prediction performance.

### 3.2 Ablation experiments

In this paper, various improvements were made to the standard YOLOv5 model. To assess whether these improvements were effective and their interactions, the causality of each improvement component was analyzed using ablation experiments (Zhu et al., 2021a). The testing results of the performance of different models using the constructed dataset of Chinese fir seedling terminal buds are shown in **Table 2**.

**Table 2:** Results of ablation experiment.

Methods	Model number	MSPH	Newconnections	Attention mechanism			TTA	Precision (%)	Recall (%)
				**CBAM**	**ECA**	**CBAM+ECA**		
YOLOv5	–	–	–	–	–	–	–	88.36 ± 0.92d	87.78 ± 0.58d
Improved model	M1	√	–	–	–	–	–	90.63 ± 1.85c (↑2.27)	90.56 ± 1.86c (↑3.96)
	M2	√	√	–	–	–	–	91.02 ± 0.96c (↑0.39)	90.98 ± 0.21c (↑1.34)
	M3	√	√	√	–	–	–	90.21 ± 0.70c (↓0.81)	90.33 ± 1.03c (↓0.65)
	M4	√	√	–	√	–	–	93.04 ± 0.69b (↑2.83)	93.45 ± 0.50b (↑3.12)
	M5	√	√	–	–	√	–	93.43 ± 0.50b (↑0.39)	93.87 ± 0.74b (↑0.42)
	M6	√	√	–	–	√	√	**95.55 ± 0.54a (↑2.12)**	**95.84 ± 0.87a (↑1.97)**

MSPH represents the micro-scale predictive head and all experimental results were tested five times by taking the mean, CBAM represents the Convolutional Block Attention Module, ECA represents the Efficient Channel Attention Module. Different letters indicate that there is significant difference (p ≤ 0.05) for one-way ANOVA and Duncan test. Bars indicate ± SD as calculated from one-way ANOVA.

Bold values means the best value among multiple data sets.

The symbol “↑” indicates an increase in the improved model’s presicion compared with original model, and the symbol “↓” indicates a decrease in the improved model’s presicion compared with that using original model.

By comparing the M1 model with the standard YOLOv5 model, it can be found that the addition of the micro-scale prediction head can effectively increase the model’s accuracy. The M1 model has a recall rate 3.96 percentage points higher than that of the standard YOLOv5, and it has a precision and a recall significantly higher than that of the standard YOLOv5 (p<0.05), indicating that the use of the micro-scale prediction head can reduce the leakage of tiny terminal buds to a certain extent. M2 is based on M1, on which new connections are added, leading to an increase by 0.39 and 1.34 percentage points in the precision and recall rates, respectively, and it has a precision and a recall significantly higher than that of the standard YOLOv5 (p<0.05). The performance of different attention modules was also tested to evaluate their effectiveness. In order to highlight the key feature information of the terminal buds and suppress the useless background information, attention modules were added to the network and the detection abilities of different modules were evaluated. When M3 model incorporates CBAM dual channel attention mechanism, the performance of the M3 model was poor, and its precision and recall rate decreased by 0.81 and 0.65 percentage points, respectively. No significant difference (p>0.05) between M3 and M2 indicates that the new microscale prediction head and the new connection method have a functional conflict with the CBAM attention module, leading to certain side effects. By replacing the CBAM attention module with ECA attention module, the M4 model has a precision rate of 93.04% and a recall rate of 93.45%, which is significantly higher (p<0.05) than the M3 model. When combining ECA and CBAM in M5 to implement the attention mechanism, although there is no significant difference (p>0.05) in performance between M5 and M4, the M5 model’s performance is better than the model using the CBAM or ECA attention module alone. Its precision and recall rates are as high as 93.43% and 93.87%, respectively. This result indicates that the attention mechanism combining ECA-Net and CBAM can improve the model’s accuracy to a greater extent, which is an ideal combination. In addition, the performance of the multi-scale test with the introduction of TTA was also tested, and the results showed that the accuracy of the model (M6) with TTA was further improved, and its precision and recall rates were 95.55% and 95.84%, respectively; there is a significant difference (p<0.05) in accuracy between M6 and other models, so we adopts the M6 model as the final version of improved YOLOv5 model in our study.

**Figure 13:**
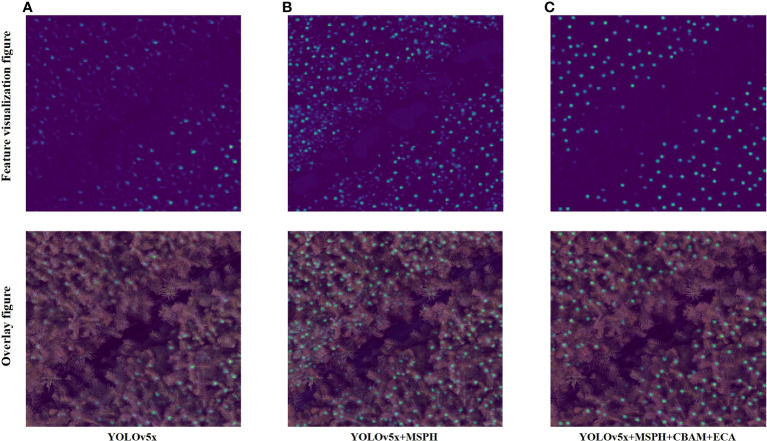
Feature visualization results. **(A)** YOLOv5, **(B)** Improved model M1, **(C)** Improved model M6.

To more intuitively display the target feature information extracted by the model, this paper visualized the features of the model before and after improvement (**Figure 13**). By comparison, it is found that the standard YOLOv5 (**Figure 13A**) can only detect the large terminal buds, but it was challenging to identify the tiny terminal buds. In contrast, the M1 model (**Figure 13B**), with the addition of the micro-scale prediction head, can detect more terminal buds. Still, it also brought more background noise, affecting terminal bud identification accuracy. For the final improved model M6 (**Figure 13C**), by introducing an attention mechanism, it can focuse on the terminal bud region to utilize more detailed features and suppress useless background information, thereby improving the recognition accuracy and efficiency of the model.

### 3.3 Comparison of recognition effect of terminal buds of Chinese fir seedlings

**Figure 14:**
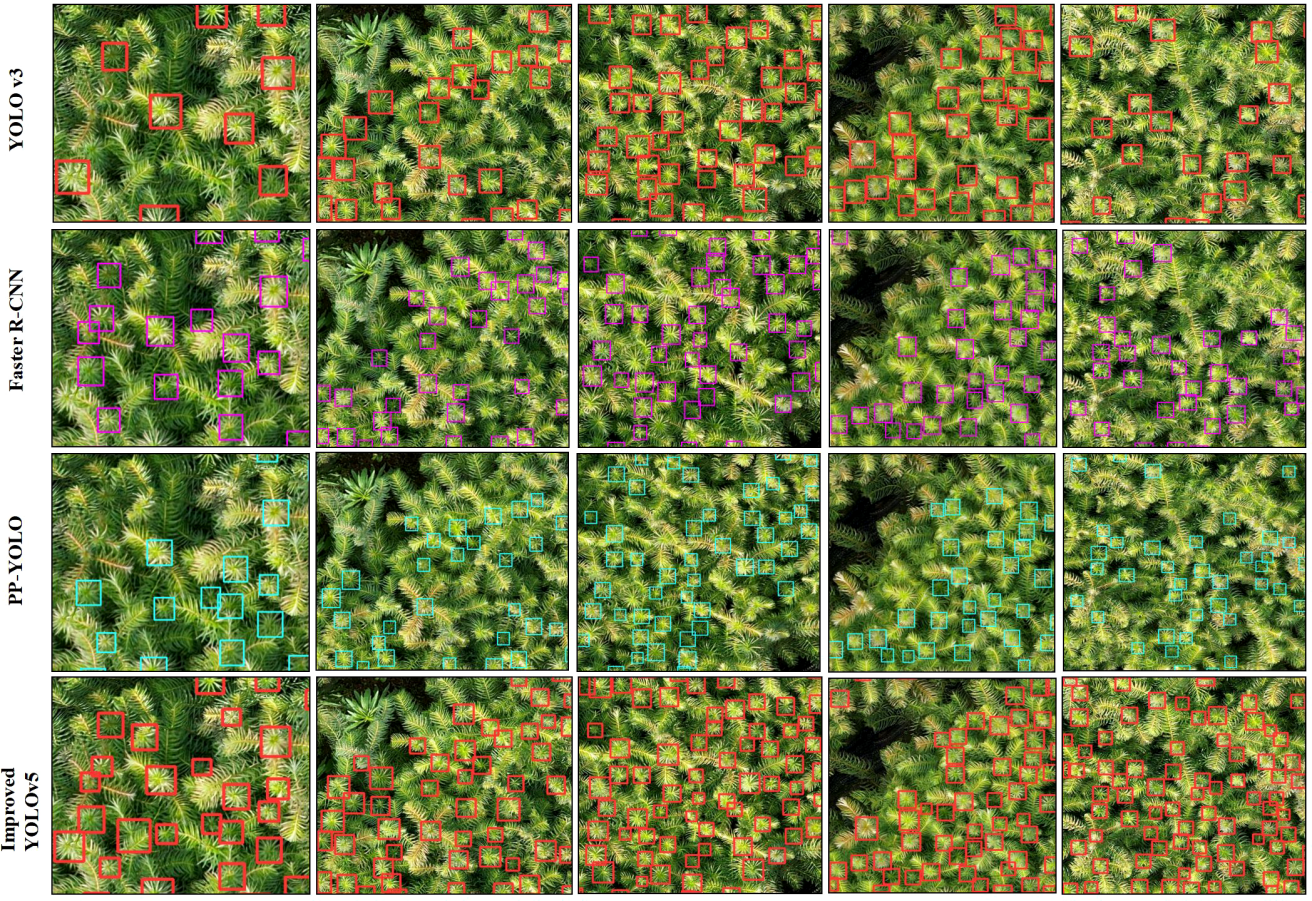
Comparison of recognition of the terminal bud of Chinese fir seedlings for different algorithms.

By comparing the improved YOLOv5 algorithm proposed in this paper with the current mainstream target detection algorithms (YOLOv3, Faster R-CNN and PP-YOLO) (**Figure 14**), it can be seen that the YOLOv3 algorithm has a poor recognition effect on large-scale and high-density small target objects. It can only effectively recognize the terminal buds with large size and apparent features, and its bounding box localization accuracy is also the worst. Faster R-CNN, a two-stage algorithm, has a different structure from the one-stage YOLOv3, and its accuracy is greatly improved compared to the YOLOv3. However, the effect of detecting tiny targets is still unsatisfactory, and there are many omissions. The improved PP-YOLO algorithm based on YOLOv3 performs better than Faster R-CNN. Although the number of terminal buds identified is still lower than the actual number, the generated prediction box boundaries are basically consistent with the actual terminal bud boundaries, and the accuracy is high. The improved YOLOv5 with the fusion attention mechanism proposed in this paper can better solve the recognition problem caused by high density, complex background and target size differences. It can be clearly seen from the figure that the improved YOLOv5 can accurately identify the terminal buds of different sizes (tiny, small, medium and large). Moreover, under the action of the dual attention mechanism (space and channel) and data augmentation, the terminal buds with high occlusion, partial defects and mutual adhesion can still be accurately detected. It can be seen that the detection performance of the improved YOLOv5 model is significantly better than that of the other three algorithms.

### 3.4 Performance comparison of different models

By comparing the quantitative evaluation indicators of the improved model and other models (**Table 3**), we can see that the YOLOv3 model has the fastest detection speed, with an FPS of 19.36. Still, its recognition effect is poor, and the precision rate, recall rate, mAP, and F1-Score are the lowest among the four. Faster R-CNN has a significant improvement in accuracy compared to YOLOv3, in which mAP and F1-Score are increased by 8.84 and 12.63 percentage points, respectively, but its detection speed is poor, with FPS only 1.14. Compared with Faster R-CNN, the accuracy rate of PP-YOLO is greatly improved and significantly higher than Faster R-CNN (p<0.05), but it still cannot meet the requirement of accurate detection of terminal buds of Chinese fir seedlings. Since the improved YOLOv5 adds a micro-scale prediction head, its calculation amount has increased. Still, its network balances the two indicators of recognition accuracy and completeness, and has the optimal recognition effect, with a precision rate of 95.55%, a recall rate of 95.84%, an mAP of 87.25%, and an F1-Score of 95.69%. Compared with the other three models, the average precision rate and F1-Score of the improved YOLOv5 are increased by 9.51-28.19 percentage points and 15.92-32.94 percentage points, respectively. In addition, the improved YOLOv5 is significantly higher(p<0.05) from other mainstream algorithms in many indicators. It can be seen that the improved YOLOv5 model can better meet the requirements of accurate identification of terminal buds of Chinese fir seedlings.

**Table 3:** Comparison of different target detection algorithms.

Model	Precision	Recall	mAP	F1-Score	FPS
YOLOv3	72.13% ± 0.89c	63.43% ± 1.06d	54.31% ± 2.69d	67.50% ± 0.62d	**19.36 ± 0.31d**
Faster R-CNN	86.13% ± 0.39b	74.92% ± 0.91c	63.15% ± 1.66c	80.13% ± 0.43c	1.14 ± 0.19c
PP-YOLO	95.44% ± 0.22a	78.56% ± 0.24b	71.33% ± 4.43b	86.18% ± 2.39b	12.53 ± 1.01b
Improved YOLOv5	**95.55% ± 0.91a**	**95.84% ± 0.36a**	**87.25% ± 1.92a**	**95.69% ± 0.61a**	7.29 ± 0.34a

Different letters indicate that there is significant difference (p ≤ 0.05) for one-way ANOVA and Duncan test. Bars indicate ± SD as calculated from one-way ANOVA.

Bold values means the best value among multiple data sets.

## 4 Discussions

Accurate and rapid acquisition of information on the number of densely planted Chinese fir seedlings is an essential issue in the current precision cultivation of Chinese fir seedlings. However, in the late stage of plant cultivation, the overlapping of lateral branches among individuals is too severe to identify the entire individual, while in the high-density planting nursery, the terminal bud of each seedling grows upward and has distinctive characteristics, which can be used as an identification feature. Therefore, this study optimized and improved its network structure based on the latest YOLOv5 model. It also constructed an improved YOLOv5 to recognize terminal buds of Chinese fir seedlings by fusing attention mechanisms and other advanced image processing methods. The results showed that the improved YOLOv5 outperformed the other three mainstream target detection models (YOLOv3, Faster R-CNN, and PP-YOLO), indicating that it is feasible to use the improved YOLOv5 model to detect and identify the terminal buds of densely-planted Chinses fir seedlings with high accuracy.

The improvement of the detection accuracy of the improved model firstly benefits from the introduction of micro-scale prediction heads. The terminal buds of Chinese fir seedlings account for a tiny proportion in the UAV image. The number of pixels in the length and width is generally between 12 and 48, which means that the three detection heads of the standard YOLOv5 model are performing high magnification (8-fold, 16-fold and 32-fold), it will lead to a large amount of loss of feature information of the tiny terminal buds (Zhao et al., 2021), resulting in a significant error of the model. The introduction of the micro-scale prediction head (4-fold the rate of downsampling) in the current enables the model to retain the feature information of the tiny terminal buds better. However, with the introduction of the micro-scale prediction head, some small background noises were also generated, and the lush lateral branches of Chinese fir seedlings complicate the background information of the terminal buds. Therefore, by adding a new attention mechanism module consisting of CBAM and ECA to the neck network, the model can focus on the key region of the terminal buds, further promoting the improvement of the model accuracy. It should be noted that the channel attention module in CBAM reduces the dimension of the feature map (Zhu et al., 2021a) by pooling operations to obtain the correlation between different channels. However, the pooling operation achieves image dimensionality reduction; it will also negatively affect the channel attention prediction, leading to poor model performance when the CBAM module is used alone. Therefore, this study replaces the channel attention module in CBAM with the ECA attention module with higher learning efficiency, which can effectively capture cross-channel interaction information without dimensionality reduction and significantly improve the model performance. In addition, due to the typical occlusion and overlapping of terminal buds of densely-planted Chinese fir seedlings and the differences in individual growth states, the generalization of the network trained using the original image data is poor. However, data augmentation can improve the diversity of target features and solve the problem of unbalanced or missing sample data (Wan et al., 2021), enhancing the robustness and generalization of the trained model to a certain extent. Finally, this study introduces the TTA multi-scale test and WBF fusion algorithm in the inference and prediction stage. A more realistic prediction result is obtained by performing multi-scale transformation of the test set images and weighted fusion of multiple prediction results with different resolutions. Other studies have also shown that this is an effective strategy to improve the detection effect of small objects (Zhu et al., 2021b).

It should be noted that although the improved YOLOv5 algorithm has significantly improved detection accuracy, its detection speed has decreased, with an FPS of only 7.29, which has not yet reached the standard of real-time detection. Hence, it is necessary to explore further the optimization of the model network parameters in the later stage. For example, it is required to promote the lightweight of the network through pruning operation, reduce the number of model parameters, and improve the computing efficiency to achieve real-time detection of the terminal buds of Chinese fir seedlings, which facilitates its deployment on mobile terminals and embedded devices, and further expands its application scope. At the same time, since the image collected by the UAV will be distorted, dislocated, and ghosted to a certain extent during the stitching process, some terminal buds cannot be well distinguished and labeled. There are still some shortcomings in researching labeled datasets. Therefore, it is necessary to continue focusing on the improvement of feature extraction algorithm and image data quality in future studies.

## 5 Conclusion

In this paper, we propose an improved YOLOv5 algorithm that integrates deep self-attention networks. The algorithm adds a new micro-scale prediction head to the standard YOLOv5 network, which can capture more feature information of tiny terminal buds. At the same time, the high-resolution shallow features in the backbone network are introduced into the feature fusion layer, which further reduces the loss of feature information of tiny terminal buds. In addition, the attention module combining CBAM and ECA attention mechanisms is also added to the feature fusion layer, which helps the model extract feature information of key regions of terminal buds in complex backgrounds, and the TTA multi-scale test and WBF fusion algorithm were used to further improve the detection ability of this method in the dense fir seedling terminal buds in UAV images. The results of this study show that the improved YOLOv5 has significantly improved the recognition accuracy compared with the standard YOLOv5. Compared with other current mainstream target detection algorithms (YOLOv3, Faster R-CNN, and PP-YOLO), the precision, recall, mean accuracy, and F1-score of the improved YOLOv5 are also improved to varying degrees. Still, the complexity of the improved YOLOv5 network is high, and the image quality needs to be improved. In the future, it is necessary to explore a high-performance, lightweight network and optimize the image quality to achieve accurate real-time detection. In summary, the improved YOLOv5 model can be applied to accurately identify of the terminal buds of densely-planted Chinese fir seedlings and target identification in high-density, multi-occlusion, and complex background scenes. It can provide technical reference for the application of consumer-grade UAVs in precision breeding, phenotype monitoring and yield prediction of Chinese fir seedlings and has specific application prospects.

## Data availability statement

The raw data supporting the conclusions of this article will be made available by the authors, without undue reservation.

## Author contributions

Conceptualization, ZY, LB, and HZ; methodology, ZY, LB, and HZ; software, ZY, LB, and HZ; validation, QG and JZ; formal analysis, SG and JW; investigation, HZ, SC, XZ, and LB; resources, XZ; data curation, ZY, LB, and HZ; writing—original draft preparation, ZY, LB, and HZ; writing—review and editing, ZY, LB, and HZ; visualization, ZY, LB, and HZ; supervision, HZ; project administration, HZ; funding acquisition, HZ. All authors have read and agreed to the published version of the manuscript.

## Funding

This work was supported by the Tibet Autonomous Region Science and Technology Plan Project Key Project (XZ202201ZY0003G), National Natural Science Foundation of China (32171818), National Natural Science Foundation of China (31901298), the Natural Science Foundation of Fujian Province (2021J01059) and Innovation and Entrepreneurship Training Program for College Students of Fujian Agriculture and Forestry University (X202210389185).

## Acknowledgments

Thanks to the GitHub open source community for the original YOLOv5 code.

## Conflict of interest

The authors declare that the research was conducted in the absence of any commercial or financial relationships that could be construed as a potential conflict of interest.

## Publisher’s note

All claims expressed in this article are solely those of the authors and do not necessarily represent those of their affiliated organizations, or those of the publisher, the editors and the reviewers. Any product that may be evaluated in this article, or claim that may be made by its manufacturer, is not guaranteed or endorsed by the publisher.
